# Conductivity Size Effect of Square Cross-Section Polycrystalline Nanowires

**DOI:** 10.3390/ma12132129

**Published:** 2019-07-02

**Authors:** Rui Li, Lan Mi, Jian Wang, Mao Mao, Wenhua Gu, Yongkai Zhu

**Affiliations:** 1 State Key Laboratory for Manufacturing Systems Engineer, Xi’an Jiaotong University, Xi’an 710049, China; 2 Food Equipment Engineering and Science, Xi’an Jiaotong University, Xi’an 710049, China; 3 School of Electronic and Optical Engineering, Nanjing University of Science and Technology, Nanjing 210094, China; 4 College of Automation Engineering, Nanjing University of Aeronautics and Astronautics, Nanjing 210016, China

**Keywords:** conductivity, size effect, polycrystalline, square cross-section

## Abstract

A theoretical model for the electrical conductivity size effect of square nanowires is proposed in this manuscript, which features combining the three main carrier scattering mechanisms in polycrystalline nanowires together, namely, background scattering, external surface scattering, as well as grain boundary scattering. Comparisons to traditional models and experiment data show that this model achieves a higher correlation with the experiment data.

## 1. Introduction

Today, the features of integrated circuits (ICs) have been reduced down to a size of 10 nm or below, thus the conductivity size effect (CSE), which can cause additional power dissipation and other side effects, has become a big concern. The CSE shows that the metallic conductivity decreases with the geometric size, which is non-trivial when the size is comparable to the mean free path of the material (39 nm for copper). However, a comprehensive theoretical model for such metal interconnects is still under study for metal interconnects in ICs, which can be defined as polycrystalline metal nanowires with a square cross-section. 

The CSE was first analyzed and applied to thin films, where the film thickness is comparable to the mean free path, yet for nanowires, the size effect is two-dimensional (wire height and width for square cross-section). Fuchs and Sondheimer (the FS model) first studied the CSE in the 1930s and pointed out that in thin films, in addition to the background scattering (BS) of the carriers in bulk materials, the external surface scattering (ESS) is a main factor causing the additional conductivity decrease due to the size limit [1,2]. Based on the ideas of the FS model for thin films, Dingle proposed a model for circular cross-section wires [3], MacDonald and Sarginson proposed a model for a rectangular cross-section [4], and Chamber proposed a model for an arbitrary cross-section [5]. 

But those theories failed to include the grain boundary scattering (GBS) for polycrystalline metal wires. As Mayadas and Shatzkes (MS)’s model pointed out in the 1970s [6], the GBS plays a dominant role in polycrystalline thin films, which has been verified experimentally [7,8,9,10,11]. The MS model was the first to combine the three major carrier scattering mechanisms in polycrystalline thin films, i.e., BS, ESS, and GBS, and achieved great success. Even in recent years, people are still relying on it to evaluate the nanowire size effect [8,9,10,11]. However, it is applicable for thin films only, and requires modification for use with nanowires. 

Unfortunately, few works have been reported for CSE modeling of nanowires. Recently, Moraga and colleagues reported a quantum mechanical model for GBS in thin films and wires [12,13,14], yet it assumed perfect external surfaces scattering. As analyzed by the FS model, inelastic scattering plays an important role in the conductivity model and requires a special parameter p for detailed discussions. Another related work found was by Dimmich and Warkusz [15], which lacks derivation details, and the results differ from ours. Marom et al. [16] studied copper wires of variable widths and heights down to 100 nm. They found that when both the width and height of the wire are larger than one third of the mean free path, its resistivity exhibits a filmlike behavior with a separate contribution to the resistivity of each small dimension, and Matthiessen’s rule can then be applied to calculate the resistivity of the wire from the known expressions for the resistivity of thin films. Moors and colleagues reported their work on the resistivity scaling and electron relaxation times in metallic nanowires in 2015 [17] and further proposed a model in 2018 [18] that does not rely on phenomenological fitting parameters. Their work deepens the understanding of the physics underneath the resistivity size effect, yet it requires knowledge of “the detailed statistical properties of grains, roughness and barrier material as well as the metallic band structure and quantum mechanical aspects of scattering and confinement”.

Similar to the MS model for thin films, it is meaningful to combine all of the three main scattering mechanisms, i.e., BS, ESS, and GBS, to model the CSE for polycrystalline nanowires. A model for circular cross-section nanowires has been reported recently by our group and shows greater consistency with experimental data [19]. In many cases, especially the metal interconnects in ICs, the nanowires are fabricated by photolithography and as a result, have a square or rectangular cross-section shape. Therefore, a special model for square cross-sections is needed, which is proposed in this manuscript. We can see that the difference in the cross-section shape leads to different boundary conditions, thus requiring a different calculation processing technique. These results also differ greatly when compared to the circular case and are not apparent unless a new analysis is employed. Table 1 shows the main differences between the available models of the CSE for comparison.

## 2. Modeling and Derivation

**Figure 1:**
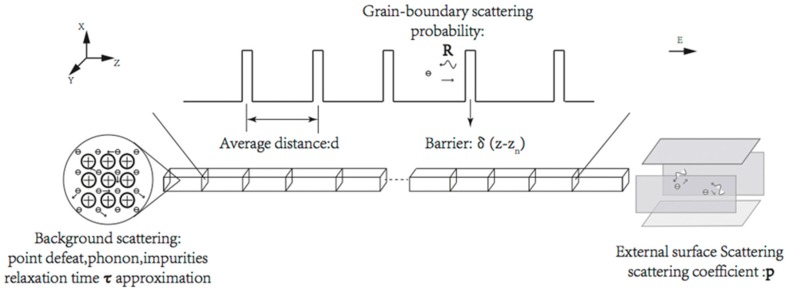
Illustration of the three scattering mechanisms in square cross-section polycrystalline nanowires.

Figure 1 illustrates the model of square cross-section nanowires. Our proposed methodology is the same as our previous work for circle cross-sections, though different boundary conditions lead to different mathematical calculations and different results. Major assumptions and simplifications of the model are listed below:

1. A square instead of a rectangular cross-section is assumed for the nanowires for simplicity, and the surface smoothness of the nanowire is not considered in this work.
2. The influence of the magnetic field is ignored here, which is reasonable for non-ferromagnetic materials. What is more, the impact of temperature variation on the resistivity is not included in the model since its relationship with cross-section shape is weak.
3. The impact of ESS is included in the model by introducing the two-dimension boundary conditions into the Boltzmann equation, as MacDonald proposed before.
4. The impact of GBS is included in the model by inserting a series of parallel planar potential barriers perpendicular to the electric current direction. The average separation *d* equals the mean grain diameter *D*, as assumed by the MS theory. These potential barriers scatter the electrons following the classic quantum mechanical scattering equations, with the potential of the barriers noted as δ(z − z_n_).

In our model, the main difference with the MS model is that a 2*d* boundary condition of square cross-sections should be applied instead of 1*d* for thin films, so we can follow almost the same derivation procedure as the MS model but with a different boundary condition to those applied in the ESS method. 

It is interesting that by further studying the MS and FS models, we can see that the CSE for a polycrystalline metal nanowire can be described in a general and simple way, as shown below in Equation (1):

*σ_w_* = *σ*_0_ − Δ*σ_g_* − Δ*σ_s_* = *σ_g_* − Δ*σ_s_*, (1)

where *σ_w_* is the conductivity of the nanowire, *σ*_0_ refers to the material bulk conductivity accounting for BS only, *σ_g_* refers to the conductivity considering both BS and GBS, Δ*σ_g_* is the conductivity decrease due to GBS, and Δ*σ_s_* is the conductivity decrease due to ESS. 

Following the same derivation as the MS model, the following equation can be obtained:

(2) $$f\left(\alpha \right)=\frac{\sigma_{g}}{\sigma_{0}}=3\left[\frac{1}{3}-\frac{1}{2}\alpha +\alpha^{2}-\alpha^{3}\ln\left(1+\frac{1}{\alpha }\right)\right],$$

where $\alpha =\frac{\lambda_{0}}{d}\frac{R}{1-R}$, *R* is the reflection probability at the grain boundary as defined by the MS theory, and $\lambda_{0}$ is the electron mean free path of bulk materials. As indicated by the MS theory, the GBS plays an important role in the resistivity size effect of polycrystalline materials, which has been verified by many experiments. For example, in 2000, Durkan and Wellan report an observation of the apparent GBS effect when the diameter of Au nanowires approaches the grain size (40~50 nm) [7]. When calculating the conductivity due to BS, the relaxation time τ is used to approximate collisions resulted from phonons, defects, as well as impurities. Now that GBS is considered, the original relaxation time requires modification.

Referring to the detailed induction in the MS theory, the total relaxation time $\tau^{*}$ is

(3) $$\frac{1}{\tau^{*}}=\frac{1}{\tau }\left(1+\frac{\alpha }{\cos\theta }\right)=\frac{1}{\tau }H\left(\theta \right),$$

with θ measured from the z axis, so $k_{z}=k_{F}\cos\theta$, where *k_F_* is the Fermi wave vector and *k_z_* is its *z* component. Substituting τ with $\tau^{*}$, the Boltzmann equation can be written as

(4) $$v_{x}\frac{\partial f_{1}}{\partial x}+v_{y}\frac{\partial f_{1}}{\partial y}+\frac{f_{1}}{\tau^{*}}=eEv_{z}\frac{\partial f_{0}}{\partial \varepsilon }.$$

Because Equation (4) is a nonlinear binary partial differential equation, one can obtain its general solutions by solving the eigen values.

Let $f^{\prime }=f_{1}-eEv_{z}\tau^{*}\frac{\partial f_{0}}{\partial \varepsilon }$. Expression Equation (4) can be rewritten as

(5) $$v_{x}\frac{\partial f^{\prime }}{\partial x}+v_{y}\frac{\partial f^{\prime }}{\partial y}+\frac{f^{\prime }}{\tau^{*}}=0.$$

**Figure 2:**
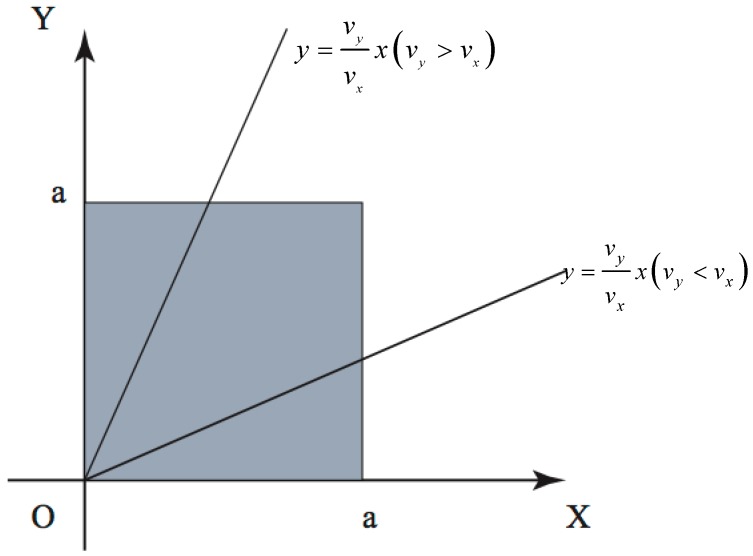
The line $y=\frac{v_{y}x}{v_{x}}$ divides the square cross-section into two parts, where the expression will be different when the slope is >1 or <1.

Equation (5) has two sub-equations: $\left\{ \begin{array}{l} \frac{dx}{v_{x}}=-\tau^{*}\frac{df^{\prime }}{f^{\prime }}\\ \frac{dy}{v_{y}}=\frac{dx}{v_{x}} \end{array}\right.$, and the solution is

(6) $$f^{\prime }=C_{1}\exp(-\frac{x}{\tau^{*}v_{x}})\;\mathrm{or}\;f^{\prime }=C_{2}\exp(-\frac{y}{\tau^{*}v_{y}})\;\mathrm{and}\;\frac{x}{v_{x}}-\frac{y}{v_{y}}=C,$$

where $C_{1}$ and $C_{2}$ are arbitrary constants. So the solution of Equation (4) is

(7) $$f_{1}=eE\tau^{*}v_{z}\frac{\partial f_{0}}{\partial \varepsilon }\left[1-e^{-\frac{x}{\tau^{*}v_{x}}}\cdot \Phi \left(\frac{x}{v_{x}}-\frac{y}{v_{y}}\right)\right],$$

or equally,

(8) $$f_{1}=eE\tau^{*}v_{z}\frac{\partial f_{0}}{\partial \varepsilon }\left[1-e^{-\frac{y}{\tau^{*}v_{y}}}\cdot \Psi \left(\frac{y}{v_{y}}-\frac{x}{v_{x}}\right)\right],$$

where Φ and Ψ can be any functions. To determine the form of Φ and Ψ, let us temporarily assume complete inelastic scattering at the nanowire surface so that $f_{1}=0$. Substitute this condition into Equations (7) and (8), and Φ and Ψ can be solved. As shown in Figure 2, the line y = v_y_*x/v_x_ divides the square cross-section into two parts.

Considering the *x = 0* boundary, when *f_1_ = 0*, we have

(9) $$f_{1}=eE\tau^{*}v_{z}\frac{\partial f_{0}}{\partial \varepsilon }\left[1-e^{-\frac{x}{\tau^{*}v_{x}}}\right],y>\frac{v_{y}}{v_{x}}x.$$

Similarly, when *y = 0*, we have

(10) $$f_{1}=eE\tau^{*}v_{z}\frac{\partial f_{0}}{\partial \varepsilon }\left[1-e^{-\frac{y}{\tau^{*}v_{y}}}\right],y<\frac{v_{y}}{v_{x}}x.$$

So the complete expression is as below.

f1=eEτ*vz∂f0∂ε[1−e−yτ*vy],y<vyvx(x−a)f1=eEτ*vz∂f0∂ε[1−ea−xτ*vx],y>vyvx(x−a)f1=eEτ*vz∂f0∂ε[1−e−yτ*vy],y<vyvxxf1=eEτ*vz∂f0∂ε[1−e−xτ*vx],y>vyvxxf1=eEτ*vz∂f0∂ε[1−ea−xτ*vx],a−y>vyvx(a−x)f1=eEτ*vz∂f0∂ε[1−ea−yτ*vy],a−y<vyvx(a−x)f1=eEτ*vz∂f0∂ε[1−e−xτ*vx],a−y>−vyvxxf1=eEτ*vz∂f0∂ε[1−ea−yτ*vy],a−y<−vyvxx

Because the distribution function in each quadrant will make the same contribution to the electric current density, we can use the expression of the first quadrant as a model to calculate the electric current density:

(11) $$J_{z}=2e{\left(\frac{m}{h}\right)}^{3}∭v_{z}f_{1}dv_{x}dv_{y}dv_{z}.$$

We have

(12) $$J_{z}=2e{\left(\frac{m}{h}\right)}^{3}\underset{0<v_{x}<v_{y}}{∭}v_{z}^{2}eE\tau^{*}\frac{\partial f_{0}}{\partial \varepsilon }\left[\begin{array}{l} 2a^{2}-2a\tau^{*}v_{y}\left(1+e^{-\frac{a}{\tau^{*}v_{x}}}\right)\\ -2a\tau^{*}v_{x}\left(1-e^{-\frac{a}{\tau^{*}v_{x}}}\right)\\ +4{\left(\tau^{*}\right)}^{2}v_{x}v_{y}\left(1-e^{-\frac{a}{\tau^{*}v_{x}}}\right) \end{array}\right]dv_{x}dv_{y}dv_{z}.$$

Let $v_{z}=v\cos\theta ,v_{x}=v\sin\theta \cos\varphi ,v_{y}=v\sin\theta \sin\varphi$. Then we get conductivity $\sigma_{w}$ in consideration of BS, ESS, and GBS:

(13) $$\frac{\sigma_{w}}{\sigma_{0}}=f\left(\alpha \right)-\frac{3}{\pi }\int_{0}^{\pi }\frac{\cos^{2}\theta \sin\theta d\theta }{H\left(\theta \right)}\int_{\pi /4}^{\pi /2}d\varphi \left[\begin{array}{l} \frac{\sin\theta \sin\varphi }{kH\left(\theta \right)}\left(1+e^{-\frac{kH\left(\theta \right)}{\sin\theta \cos\varphi }}\right)+\\ \frac{\sin\theta \cos\varphi }{kH\left(\theta \right)}\left(1-e^{-\frac{kH\left(\theta \right)}{\sin\theta \cos\varphi }}\right)-\\ \frac{2\sin^{2}\theta \sin\varphi \cos\varphi }{k^{2}H^{2}\left(\theta \right)}\left(1-e^{-\frac{kH\left(\theta \right)}{\sin\theta \cos\varphi }}\right) \end{array}\right],$$

where $f\left(\alpha \right)=3\left[\frac{1}{3}-\frac{1}{2}\alpha +\alpha^{2}-\alpha^{3}\ln\left(1+\frac{1}{\alpha }\right)\right]$, $k=a/\lambda_{0}$, $H=1+\alpha /\sqrt{\left(1-1/t^{2}\right)}$. Making a further substitution, t=1/sinθ, then $d\theta =-{\left(t\sqrt{t^{2}-1}\right)}^{-1}dt$, and we get

(14) $$\frac{\sigma_{w}}{\sigma_{0}}=f\left(\alpha \right)-\frac{6}{\pi k}\int_{1}^{\infty }\frac{1}{H^{2}}\left(\frac{1}{t^{3}}-\frac{1}{t^{5}}\right){\left(t^{2}-1\right)}^{-\frac{1}{2}}dt\int_{\pi /4}^{\pi /2}d\varphi \left[\begin{array}{l} \sin\varphi \left(1+e^{-\frac{kHt}{\cos\varphi }}\right)+\\ \cos\varphi \left(1-e^{-\frac{kHt}{\cos\varphi }}\right)-\\ \frac{2\sin\varphi \cos\varphi }{kHt}\left(1-e^{-\frac{kHt}{\cos\varphi }}\right) \end{array}\right].$$

As Equation (1) shows, the total conductivity $\sigma_{w}$ is the difference of bulk conductivity $\sigma_{0}$ subtracting $\Delta \sigma_{g}$ and $\Delta \sigma_{s}$, resulting from GBS and ESS, respectively. It matches the form

(15) $$\sigma_{w}=\sigma_{0}-\Delta \sigma_{g}-\Delta \sigma_{s}.$$

For comparison with Equation (14), the final expression of the conductivity reduction given by Gu and Xue for circular cross-section polycrystalline nanowires is

(16) σwσ0=f(α)−12πk(1−p)∑v=0∞pv∫1∞(−1H2)t−5t2−1dt×∫0π2dψ{exp[−(v+1)Hktsinψ]−exp(−vHktsinψ)}sinψ,

and the final expression of the MS theory is

(17) $$\frac{\sigma_{f}}{\sigma_{0}}=f(\alpha )-\frac{6\left(1-p\right)}{\pi k}\int_{0}^{\frac{\pi }{2}}d\phi \int_{1}^{\infty }\left(\begin{array}{l} \frac{\cos^{2}\phi }{H^{2}\left(t,\phi \right)}\left(\frac{1}{t^{3}}-\frac{1}{t^{5}}\right)\\ \times \frac{1-\exp\left[-ktH\left(t,\phi \right)\right]}{1-p\exp\left[-ktH\left(t,\phi \right)\right]} \end{array}\right)dt.$$

We can see that Equation (14) has a very similar form to Equations (16) and (17), and the main difference lies in the ratio of elastic scattering, *p*. It is because complete inelastic scattering at the nanowire external surface was assumed in the above calculations, which means *p = 0*. Considering the part of elastic scattering, similar to Equations (16) and (17), the final expression of the conductivity in square cross-section polycrystalline nanowires can be written as

(18) $$\frac{\sigma_{w}}{\sigma_{0}}=f\left(\alpha \right)-\frac{6\left(1-p\right)}{\pi k}\int_{1}^{\infty }\frac{1}{H^{2}}\left(\frac{1}{t^{3}}-\frac{1}{t^{5}}\right){\left(t^{2}-1\right)}^{-\frac{1}{2}}dt\int_{\pi /4}^{\pi /2}d\varphi \left[\begin{array}{l} \sin\varphi \left(\frac{1+e^{-\frac{kHt}{\cos\varphi }}}{1+pe^{-\frac{kHt}{\cos\varphi }}}\right)+\\ \cos\varphi \left(\frac{1-e^{-\frac{kHt}{\cos\varphi }}}{1-pe^{-\frac{kHt}{\cos\varphi }}}\right)-\\ \frac{2\sin\varphi \cos\varphi }{kHt}\left(\frac{1-e^{-\frac{kHt}{\cos\varphi }}}{1-pe^{-\frac{kHt}{\cos\varphi }}}\right) \end{array}\right].$$

Equation (18) is the final expression of the CSE for square cross-section polycrystalline nanowires. When p=0, it is easy to verify that Equation (18) will degenerate to Equation(14); while if p=1, then it degenerates to f(α). In addition, if we let R=0, or not considering the GBS, Equation (18) will be identical to MacDonald’s equation, which is reasonable.

## 3. Discussion

**Figure 3:**
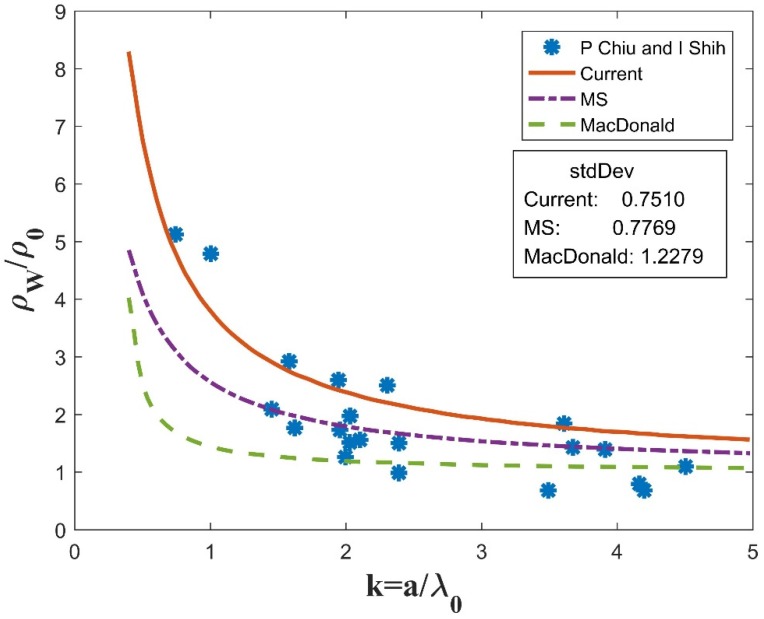
Comparison between numerical simulations and experiment data for Bi nanowires, with parameters p=0.5,R=0.5,C=1.

**Figure 4:**
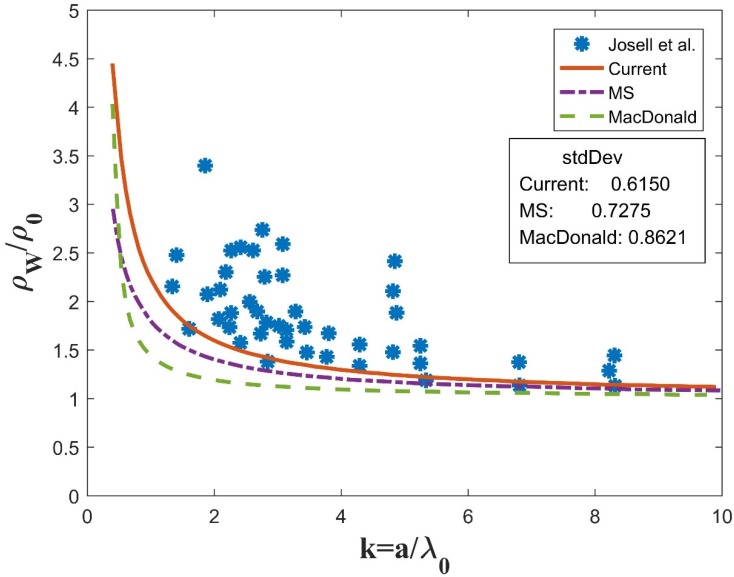
Comparison between numerical simulations and experiment data for Ag with parameters p=0.5,R=0.3,C=1.

**Figure 5:**
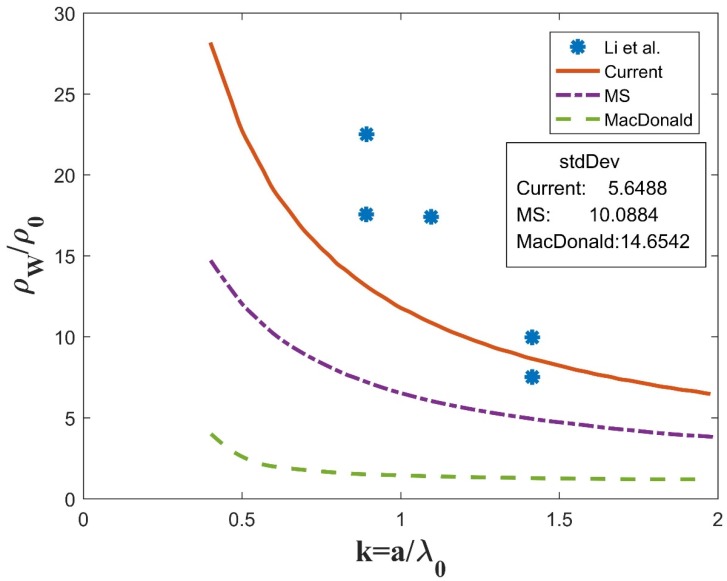
Comparison between numerical simulations and experiment data for ErSi_2_ with parameters p=0.6,R=0.8,C=1.

Unfortunately, Equation (18) cannot be solved analytically. A MATLAB program was coded to obtain its numerical solutions. Experiment data of different nanowires was collected for comparison with the current model (noted as “current”) as well as the traditional models (MS and McDonald).The same parameters (p, R, C) are used for the simulation of all three models, i.e., current, MS, and McDonald, as labeled in Figure 3, Figure 4 and Figure 5.

In Figure 3, the Bismuth experiment data points are denoted by “*”, as adopted from Figure 5 in [20], and curves for three theoretical models are also plotted for comparison. The horizontal axis *k* is the normalized side length of the square cross-section, which is equal to the ratio of the actual side length, *a*, to the mean free path, *λ_0_*. The vertical axis is the ratio of the nanowire resistivity *ρ_w_* to the bulk resistivity *ρ_0_*. The parameter values are adopted from the experiment source article as: p=0.5,R=0.5,C=1. The parameters (p, R, C) are the same as in MS and McDonald. As we can see, the current model gives higher resistivity values than the MS and McDonald models, which is reasonable since it takes into account all of the three scattering mechanisms. In addition, the standard deviation of the curve fitting was calculated to describe the degree of match of the different models to the experiment data. It is defined as the mean squared error (MSE) between the experiment data and theoretical values, and is described as follows:

(19) $$R=\sqrt{\frac{\sum_{i=1}^{n}\left(T(i)-A(i)\right)}{n}},$$

where R is the standard deviation; T(i) is the theoretical value; and A(i) is the related experiment data. The smaller the standard deviation is, the better the simulation curve fits with the experiment data. As Figure 3 shows, the current model has a standard deviation of 0.7510, which is smaller than the MS (0.7769) and McDonald (1.2279) models. The McDonald model has the largest standard deviation, implying that GBS contributes more than ESS to the CSE in polycrystalline nanowires.

Ag and ErSi_2_ experiment data was also collected for comparison. Figure 4 illustrates the Ag experiment data coming from Figure 5 in [21] by Josell et al. Specific parameters are set according to the experiment data source document as p=0.5,R=0.3,C=1. The current model again gives higher resistivity than the MS and McDonald models. Moreover, it fits better with the experiment data, with the smallest standard deviation of 0.6150 vs. 0.7275 for the MS curve and 0.8621 for the McDonald curve.

Figure 5 shows the data for ErSi_2_ nanowires with the parameters p=0.6,R=0.8,C=1. The experimental data was adopted from Figure 4 in [22]. Again, the current model shows higher resistivity than the MS and McDonald models and is closer to the experiment data (the standard deviation is 5.6488 vs. 10.0884 for MS and 14.6542 for McDonald) [22]. Due to limited original experiment data points (only 5), the MSE of ErSi2 is one order of magnitude larger than that of Bi and Ag nanowires for all models.

From the above model derivation process, we can see that in principle, this model is not limited to metal nanowires but could be extended to semiconductor nanowires. Further study is needed for semiconductor nanowires.

## 4. Conclusions

In conclusion, a complete model for polycrystalline metal nanowires with square cross-sections is proposed, which includes three distinct scattering mechanisms—background scattering, external surface scattering, and grain boundary scattering. Though the model is still a semi-classic one, it can be a precise model for the nanowire CSE and is especially useful for interconnects in ICs. Comparison with previous models, i.e., the MacDonald model and MS model, shows that our proposed model matches better with the experimental data.

## Figures and Tables

**Table 1:** Comparison of available models with corresponding scattering mechanism.

Theory		Scattering			Cross-Section
		BS	ESS	GBS	
Film	FS	√	√		*
	MS	√	√	√	*
Nanowires	Dingle	√	√		Circle
	Xue&Gu	√	√	√	
	MacDonald	√	√		Square
	Present	√	√	√	

FS: Fuchs and Sondheimer; MS: Mayadas and Shatzkes; BS: background scattering; ESS: external surface scattering; GBS: grain boundary scattering.

## References

[1] Fuchs K. (1938). The Conductivity of Thin Metallic Films According to the Electron Theory of Metals. Proc. Camb. Philos. Soc..

[2] Vasileska D., Raleva K., Goodnick S.M. (2010). Electrothermal Studies of FD SOI Devices That Utilize a New Theoretical Model for the Temperature and Thickness Dependence of the Thermal Conductivity. IEEE Trans. Electron Devices.

[3] Dingle R.B., Lawrence B.W. (1950). The Electrical Conductivity of Thin Wires. Proc. R. Soc. Lond. Ser. A Math. Phys. Sci..

[4] MacDonald D.K.C., Sarginson K. (1950). Size Effect Variation of the Electrical Conductivity of Metals. Proc. R. Soc. Lond. Ser. A Math. Phys. Sci..

[5] Chambers R.G. (1950). The Conductivity of Thin Wires in a Magnetic Field. Proc. R. Soc. Lond. Ser. A Math. Phys. Sci..

[6] Madayas A.F., Shatzkes M. (1970). Electrical-Resistivity Model for Polycrystalline Films: The Case of Arbitrary Reflection at External Surfaces. Phys. Rev. B.

[7] Durkan C., Welland M.E. (2000). Size Effects in the Electrical Resistivity of Polycrystalline Nanowires. Phys. Rev. B.

[8] Wu W., Brongersma S.H., Hove M.V., Meax K. (2004). Influence of Surface and Grain-Boundary Scattering on the Resistivity of Copper in Reduced Dimensions. Appl. Phys. Lett..

[9] Steinhögl W., Schindler G., Steinlesberger G., Traving M., Engelhardt M. (2005). Comprehensive Study of the Resistivity of Copper Wires with Lateral Dimensions of 100nm and Smaller. J. Appl. Phys..

[10] Lim J.W., Isshiki M. (2006). Electrical Resistivity of Cu Films Deposited by Ion Beam Deposition: Effects of Grain Size, Impurities, and Morphological Defect. J. Appl. Phys..

[11] Karim S., Ensinger W., Cornelius T.W., Neumann R. (2008). Investigation of Size Effects in the Electrical Resistivity of Sinle Electrochemically Fabricated Gold Nanowires. Phys. E.

[12] Moraga L., Arenas C., Henriquez R., Solis B. (2015). The Effect of Surface Roughness and Grain-Boundary Scattering on the Electrical Conductivity of Thin Metallic Wires. Phys. Status Solidi B.

[13] Arenas C., Henriquez R., Moraga L., Muñoz E., Munoz R.C. (2015). The Effect of Electron Scattering from Disordered Grain Boundaries on the Resistivity of Metallic Nanostructures. Appl. Surf. Sci..

[14] Moraga L., Henriquez R., Solis B. (2015). Quantum Theory of the Effect of Grain Boundaries on the Electrical Conductivity of Thin Films and Wires. Phys. B.

[15] Dimmich R., Warkusz F. (1986). Electrical Conductivity of Thin Wires. Act. Passiv. Electron. Compon..

[16] Marom H., Mullin J., Eizenberg M. (2006). Size-Dependent Resistivity of Nanometric Copper Wires. Phys. Rev. B.

[17] Moors K., Soree B., Tokei Z. (2014). Resistivity Scaling and Electron Relaxation Times in Metallic Nanowires. J. Appl. Phys..

[18] Moors K., Sorée B., Magnus W. (2017). Resistivity Scaling in Metallic Thin Films and Nanowires due to Grain Boundary and Surface Roughness Scattering. Microelectron. Eng..

[19] Xue W.H., Gu W.H. (2016). Conductivity Size Effect of Polycrystalline Metal Nanowires. AIP Adv..

[20] Chiu P., Shih I. (2004). A Study of the Size effect on the Temperature-Dependent Resistivity of Bismuth Nanowires with Rectangular Cross-Sections. Nanotechnology.

[21] Josell D., Burkhard C., Li Y., Cheng Y.-W., Keller R.R., Witt C.A., Kelley D.R., Bonevich J.E., Baker B.C., Moffat T.P. (2004). Electrical Properties of Superfilled Submicrometer Silver Metallizations. J. Appl. Phys..

[22] Li Z.G., Long S.B., Wang C.S., Liu M., Wu W.G., Hao Y.L., Zhao X.W. (2006). Resistivity Measurements of Self-Assembled Epitaxially Grown Erbium Silicide Nanowires. J. Phys. D Appl. Phys..

